# riboviz 2: a flexible and robust ribosome profiling data analysis and visualization workflow

**DOI:** 10.1093/bioinformatics/btac093

**Published:** 2022-02-14

**Authors:** Alexander L Cope, Felicity Anderson, John Favate, Michael Jackson, Amanda Mok, Anna Kurowska, Junchen Liu, Emma MacKenzie, Vikram Shivakumar, Peter Tilton, Sophie M Winterbourne, Siyin Xue, Kostas Kavoussanakis, Liana F Lareau, Premal Shah, Edward W J Wallace

**Affiliations:** Department of Genetics, Rutgers University, Piscataway, NJ 08854-8082, USA; Institute for Cell Biology and SynthSys, School of Biological Sciences, The University of Edinburgh, Edinburgh EH9 3BF, UK; Department of Genetics, Rutgers University, Piscataway, NJ 08854-8082, USA; EPCC, The University of Edinburgh, Edinburgh EH8 9BT, UK; Center for Computational Biology, University of California, Berkeley, CA 94720, USA; Institute for Cell Biology and SynthSys, School of Biological Sciences, The University of Edinburgh, Edinburgh EH9 3BF, UK; EPCC, The University of Edinburgh, Edinburgh EH8 9BT, UK; Institute for Cell Biology and SynthSys, School of Biological Sciences, The University of Edinburgh, Edinburgh EH9 3BF, UK; Department of Bioengineering, University of California, Berkeley, CA 94720, USA; Department of Genetics, Rutgers University, Piscataway, NJ 08854-8082, USA; Institute for Cell Biology and SynthSys, School of Biological Sciences, The University of Edinburgh, Edinburgh EH9 3BF, UK; Institute for Cell Biology and SynthSys, School of Biological Sciences, The University of Edinburgh, Edinburgh EH9 3BF, UK; EPCC, The University of Edinburgh, Edinburgh EH8 9BT, UK; Center for Computational Biology, University of California, Berkeley, CA 94720, USA; Department of Bioengineering, University of California, Berkeley, CA 94720, USA; Department of Genetics, Rutgers University, Piscataway, NJ 08854-8082, USA; Institute for Cell Biology and SynthSys, School of Biological Sciences, The University of Edinburgh, Edinburgh EH9 3BF, UK

## Abstract

**Motivation:**

Ribosome profiling, or Ribo-seq, is the state-of-the-art method for quantifying protein synthesis in living cells. Computational analysis of Ribo-seq data remains challenging due to the complexity of the procedure, as well as variations introduced for specific organisms or specialized analyses.

**Results:**

We present riboviz 2, an updated riboviz package, for the comprehensive transcript-centric analysis and visualization of Ribo-seq data. riboviz 2 includes an analysis workflow built on the Nextflow workflow management system for end-to-end processing of Ribo-seq data. riboviz 2 has been extensively tested on diverse species and library preparation strategies, including multiplexed samples. riboviz 2 is flexible and uses open, documented file formats, allowing users to integrate new analyses with the pipeline.

**Availability and implementation:**

riboviz 2 is freely available at github.com/riboviz/riboviz.

## 1 Introduction

Ribo-seq quantifies the ‘translatome’ of actively translated RNAs in cells (Ingolia *et al.*, 2009). Ribo-seq combines high-throughput sequencing with nuclease footprinting of ribosomes to identify the location of ribosomes across the transcriptome at codon-level resolution. Ribo-seq is often combined with RNA-seq to quantify post-transcriptional regulation and also enables quantitative mechanistic insight into the movement of ribosomes along RNA. Specialized pipelines are needed for Ribo-seq data, covering preprocessing, read mapping, gene-specific and codon-specific quantification and other downstream analyses (Li *et al.*, 2020). We previously developed riboviz as an analysis and visualization framework for Ribo-seq data (Carja *et al.*, 2017). Here, we present a significantly expanded and reworked version: riboviz 2.

## 2 Materials and methods

The riboviz 2 pipeline is implemented via Nextflow (Di Tommaso *et al.*, 2017; Jackson *et al.*, 2021) to process multiple samples from an experiment in a single command-line call. All run-specific parameters are specified by the user in a single YAML-format configuration file, documented at github.com/riboviz/riboviz. Users may also utilize a graphical user interface (GUI) to aid in the generation of this configuration file. The configuration file facilitates reproducible and transparent analyses, and allows the pipeline to run on various computing systems. riboviz 2 invokes both publicly available tools [e.g. cutadapt (Martin, 2011), HISAT2 (Kim *et al.*, 2015), UMI-tools (Smith *et al.*, 2017)], and custom Python and R scripts for data parsing and visualization.

**Fig. 1.**
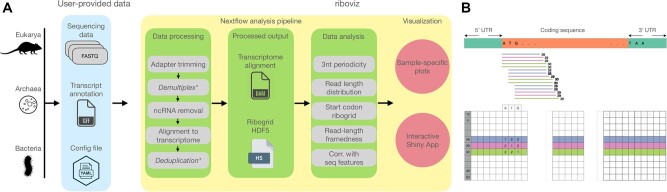
riboviz pipeline and data structures. (**A**) riboviz takes in user-provided sequencing, transcript annotation, and configuration files, processes the datasets and generates two major output—transcriptome-specific BAM file and a ribogrid file. These outputs are used to generate sample-specific analyses and summaries, which can be visualized as both static figures and in an interactive R/Shiny application. (**B**) Structure of the ribogrid file format. Ribogrid is a complete representation of transcript-specific ribosome-footprint data in an H5 file format. Each row indicates reads of a particular length and each column indicates the position of the 5ʹ-end of a footprint. *Optional

**Fig. 2.**
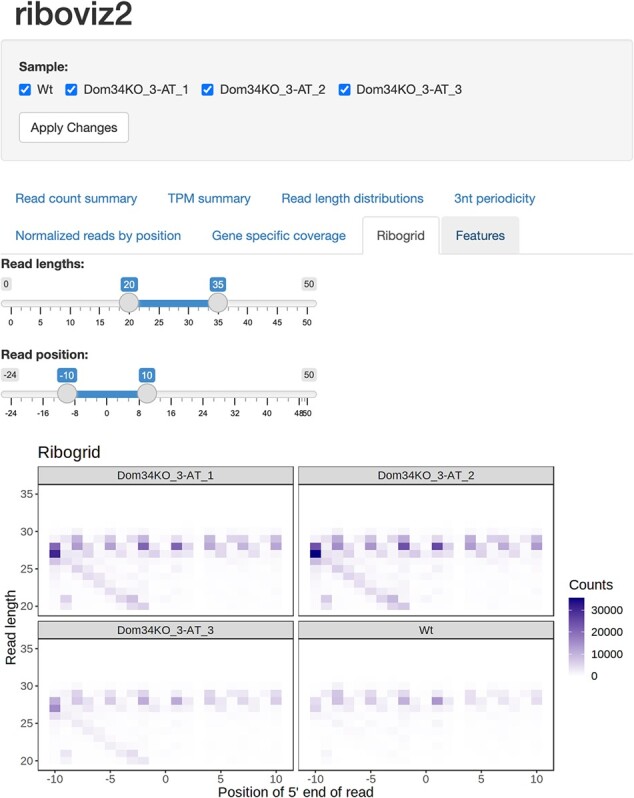
The riboviz 2 data visualization application is powered by R and Shiny and allows users to view various aspects of their data in an interactive manner in a web browser. Shown is an example visualization using data from Guydosh and Green (2014). This dataset is also part of our example datasets repository (https://github.com/riboviz/example-datasets).

The riboviz 2 workflow (Fig. 1A) starts with preprocessing of Ribo-seq data in FASTQ format, including adapter trimming and removing reads mapping to user-supplied contaminant sequences such as rRNA. Following preprocessing, the remaining reads are aligned to the relevant sequences as defined by user-provided FASTA and GFF3 files. Due to differences in ribosome structure and Ribo-seq protocols, the appropriate strategy for assigning reads to the codon at the ribosomal active site varies between species, e.g. Ribo-seq reads from eukaryotes and bacteria are mapped relative to the 5ʹ- and 3ʹ-end, respectively (Mohammad *et al.*, 2019). riboviz 2 allows the user to map relative to either end of the read by specifying the displacement separately for each desired read length.

riboviz 2 provides outputs typical to Ribo-seq in standard file formats, including the aligned reads in BAM format and number of read counts by read length in text format. We provide a ribogrid’ intermediate data file in H5 format that contains one aligned read count matrix per transcript, organized by both 5ʹ position and read length. These counts are a sufficient statistic for most downstream analyses, in that the only information used from the raw alignments is the count by both position and length. Documentation and accessor functions for this ribogrid H5 file format enable the future addition of custom analysis functions.

riboviz 2 automatically outputs visualizations commonly used in publications describing Ribo-seq experiments, both for quality control to confirm that the experiment successfully recovered ribosome footprints, and as a valuable tool for analysis. These include read length distributions, proportion of reads mapping to the primary, +1, and +2 reading frames per gene, and metagene plots showing three-nucleotide periodicity. riboviz 2 directly visualizes the aligned read count matrix, with a heatmap of the footprint counts arranged by both 5ʹ position and read length (Fig. 1B). These ‘ribogrid’ plots are a rich way to read out mechanistic details of Ribo-seq data such as read frame (Lareau *et al.*, 2014). For each processed sample, the various plots output by riboviz 2 are combined into static HTML file as an overall visual summary. riboviz 2 can compare codon-specific ribosome densities to features or measures expected to correlate with elongation rates, such as tRNA gene copy numbers, and compare gene-specific features (such as codon usage metrics) to gene-level quantifications of ribosome density. In addition to these static visualizations on a per sample basis, riboviz 2 allows users to interactively visualize all of their data in an R/Shiny based web application (Fig. 2). The Shiny app is particularly useful for comparing results across control and treatment samples. Users can adjust interactive versions of the static plots already provided as well as view gene-level statistics such as read distribution along a specific gene.

## 3 New features and advantages

### Flexibility across organisms

riboviz 2 can be used on any organism for which a transcriptome FASTA and GFF3 file can be constructed, making it a valuable tool for users studying either model or non-model organisms. This is an advantage for riboviz 2 compared to other GUI or command-line based tools that are limited to a set of organisms or require sequence annotations to be downloaded from a specific database (Liu *et al.*, 2020; Perkins *et al.*, 2019; Verbruggen *et al.*, 2019; Wang *et al.*, 2018). The user is responsible for supplying a FASTA file appropriate to their biological question, e.g. using a published annotation to define spliced transcripts including untranslated regions, or a ‘padded ORFeome’ that contains fixed-width extensions to a set of open reading frames (ORFs) of interest. The user must also supply a file in GFF3 format that specifies the positions of ORFs within the transcripts. Example configuration files to run riboviz 2 on diverse datasets that span the major domains of life (Archaea, Bacteria and Eukarya), with matched transcriptome and contaminant files, are shared at (github.com/riboviz/example-datasets). These files may be used to reproduce analyses, or adapted to analyze new datasets.

### Flexible end-to-end data processing workflow

Another advantage of riboviz 2 is that it provides a comprehensive workflow starting from raw reads and ending with publication-quality figures. Many pipelines require input that has either already been preprocessed or aligned [see Li *et al.* (2020) for a summary of the functionality of other pipelines]. Instead, riboviz 2 provides comprehensive data preprocessing (e.g. adapter trimming) and read alignment by interfacing to cutadapt (Martin, 2011) and HISAT2 (Kim *et al.*, 2015). riboviz 2 is also flexible to variations in library preparation. To the best of our knowledge, riboviz 2 is the only Ribo-seq pipeline which is prebuilt to handle multiplexed libraries or unique molecular identifiers. Following read alignment, riboviz 2 uniquely invokes an (optional) script to trim non-templated 5ʹ mismatches added by some viral reverse transcriptases (Wulf *et al.*, 2019), which otherwise leads to inaccurate quantification of read frame. riboviz 2 requires no knowledge of Python or R to take advantage of the riboviz 2 functionality, unlike many other tools (Backman and Girke, 2016; Lauria *et al.*, 2018). As riboviz 2 is implemented as a Nextflow workflow going from raw data to visualization while requiring only a single configuration YAML file, reproducing an analysis does not require independently running various tools.

### Flexible and documented data outputs

A major goal of a Ribo-seq analysis pipeline is to enable further downstream analyses of Ribo-seq data, such as differential expression analysis and identification of ribosome pausing sites. riboviz 2 consolidates the data into outputs that are suitable for downstream analysis, such as aligned read count matrices in the ribogrid H5 file. riboviz 2 aggregates raw counts per transcript into a format which can be used as input to tools such as DESeq2 (Love *et al.*, 2014), and provides per-ORF translation values in transcripts per million (TPM).

Overall, riboviz 2 is a flexible, documented and carefully engineered open-source workflow for Ribo-seq analysis and visualization.

## Funding

This work was supported by the Biotechnology and Biological Sciences Research Council [BB/S018506/1 to E.W.J.W.]; the Wellcome Trust [208779/Z/17/Z to E.W.J.W., 204804/Z/16/Z to The University of Edinburgh]; the National Science Foundation [DBI 1936046 to P.S., DBI 1936069 to L.F.L.]; the National Institutes of Health [R35 GM124976 and subcontracts from R01 DK056645, R01 DK109714, R01 DK124369 to P.S., R01 GM132104 to L.F.L]; and start-up funds from the Human Genetics Institute of New Jersey at Rutgers University to P.S.

*Conflict of Interest*: none declared.

## Data Availability Statement

The source code and data underlying this article are available in figshare, at https://dx.doi.org/10.6084/m9.figshare.12624200. The development version of the source code is also available at https://github.com/riboviz/riboviz, where we welcome user comments and contributions.
